# Goals of Surgical Interventions in Youths Receiving Palliative Care

**DOI:** 10.1001/jamanetworkopen.2024.44072

**Published:** 2024-11-08

**Authors:** Danielle I. Ellis, Li Chen, Samara Gordon Wexler, Madeline Avery, Tommy D. Kim, Amy J. Kaplan, Emanuele Mazzola, Cassandra Kelleher, Joanne Wolfe

**Affiliations:** 1 Department of Surgery, Massachusetts General Hospital, Harvard Medical School, Boston; 2 Division of Psychosocial Oncology and Palliative Care, Boston Children’s Hospital/Dana Farber Cancer Institute, Harvard Medical School, Boston, Massachusetts; 3 Department of Data Science, Dana Farber Cancer Institute, Harvard Medical School, Boston, Massachusetts; 4 Department of Pediatrics, Massachusetts General Hospital, Harvard Medical School, Boston

## Abstract

**Importance:**

Most youths receiving palliative care undergo many surgical interventions over their lifetimes. The intended purposes of interventions in the context of goals of care are not commonly articulated.

**Objective:**

To describe the goals and purposes of surgical intervention in youths receiving palliative care and propose a framework discussing intervention using goal-oriented language.

**Design, Setting, and Participants:**

This retrospective cohort analysis was conducted among a subset of patients enrolled between April 2017 and March 2021 in a prospective multicenter cohort study of youths receiving palliative care (the Pediatric Palliative Care Research Network’s Shared Data and Research [SHARE] Study). Patients younger than 30 years receiving palliative care services were eligible for inclusion in SHARE, and all enrolled at Boston Children’s Hospital/Dana Farber Cancer Institute, a SHARE site, were included in this study. Goals and purposes of all surgical interventions from the time of diagnosis through the present were abstracted from patient records. A goal and purpose framework was generated using a hybrid deductive-inductive approach based on established goals-of-care frameworks and the clinical context of surgical interventions. Data were analyzed in September 2023.

**Main Outcomes and Measures:**

Primary outcomes included goals and purposes of surgical interventions performed in the study population.

**Results:**

Among 197 youths receiving palliative care (mean [SD] age at palliative care start, 8.01 [7.53] years; 108 male [54.8%]; 6 Asian [3.0%], 12 Black [6.1%], 129 White [65.5%], and 16 with >1 race [8.1%]; 27 Hispanic [13.7%] and 142 not Hispanic [72.1%]), almost all individuals (189 youths [95.9%]) underwent at least 1 surgical intervention (mean [SD] 17.5 [16.3] interventions; median [IQR] 13 [5-22] interventions). Of 3331 surgical interventions, there were 878 interventions (26.5%) conducted with the goal of life extension, 1229 interventions (37.1%) conducted for life enhancement, and 79 interventions (2.4%) conducted for both goals; the remaining 1130 interventions (34.1%) held neither goal. Most interventions were performed with the purpose of diagnosis (1092 interventions [32.9%]) or cure and repair (1055 interventions [31.8%]), with fewer performed for the purpose of placing or maintaining assistive technology (696 interventions [21.0%]) or for supportive (434 interventions [13.1%]) or temporizing (39 interventions [1.2%]) purposes. Patients with cardiovascular disease and cancers constituted approximately half (592 patients [56.1%]) of those undergoing curative or repair interventions, whereas youths with neurologic or genetic conditions constituted approximately half (244 patients [56.2%]) of those undergoing supportive interventions.

**Conclusions and Relevance:**

In this cohort study, nearly all youths underwent surgical intervention, and the purposes of intervention differed by serious illness type. These findings suggest that conversations centered on a proposed framework concerning goals and purposes of surgical intervention may facilitate goal-concordant, high-quality care for youths with serious illness.

## Key Points

**Question:**

What are the goals and purposes of surgical intervention in youths with serious illnesses receiving palliative care?

**Findings:**

In this cohort study of 197 youths receiving palliative care, interventions were performed with goals of helping youths feel better and live longer and for the purposes of diagnosis, cure and repair, and assistive technology more so than for symptom support or as a temporizing measure. Youths with more acute illnesses underwent the most curative and repair interventions (particularly in the early postdiagnosis period), whereas those with more chronic illnesses underwent most of the supportive interventions.

**Meaning:**

These findings suggest that conversations using the proposed framework concerning goals and purposes of surgical intervention may facilitate goal-concordant, high-quality care for youths with serious illness.

## Teaser

This cohort study investigates goals and purposes of surgical interventions among youths receiving palliative care.

## Introduction

Children with serious illness often undergo surgical intervention, and pediatric surgeons play an essential role in counseling and treating these children.^1,2^ That is especially true for one population of children with serious illness in particular: those receiving pediatric palliative care (PPC).^3,4^ Previous literature has shown that most children (81%) receiving palliative care undergo at least 1 surgical intervention after serious illness diagnosis.^5^ Generally, there are limited data on the goals of surgical intervention across populations,^6^ including for children with serious illness. Existing literature on the intersection of pediatric surgery and palliative care has focused on interventions performed for symptom support or at the end of life rather than on the goals of interventions throughout the illness trajectory.^7,8^

High-quality care in serious illness is predicated on goal concordance, and goal concordance is predicated on high-quality communication.^9^ Understanding the reason an intervention is being recommended, what the intervention is meant to accomplish (ie, its intended purpose), and the likelihood that the intervention will align with the goals of care are essential to achieving goal concordance among teams, patients, and families. In the absence of literature defining the intended purpose of surgical interventions over the lifetimes of children with seriously illness and of evidence-based frameworks to guide goal-grounded communication with families and between teams, we conducted a cohort study of youths with seriously illness receiving PPC to assess the primary goals and intended purposes of surgical interventions these patients underwent after serious illness diagnosis and throughout their illness trajectories. Based on these findings, we proposed a preliminary framework grounded in the purpose of intervention and mirroring goals-of-care language to guide communication about surgical intervention in youths with serious illness.

## Methods

### Study Design and Population

We performed a retrospective cohort analysis of surgical interventions in youths from Boston Children’s Hospital/Dana Farber Cancer Institute (BCH/DFCI) who were enrolled in a multicenter prospective cohort study (the Pediatric Palliative Care Research Network’s Shared Data and Research [SHARE] Study) conducted at 7 children’s hospitals (Children’s Hospital of Philadelphia, BCH/DFCI, Seattle Children’s, Children’s Hospital and Clinics of Minnesota, Akron Children’s Hospital, Children’s of Alabama, and Texas Children’s).^10^ Patients younger than 30 years receiving PPC services were eligible for inclusion in SHARE. Data as a part of SHARE were collected between April 2017 and March 2021. Data for this study were abstracted in 2023 and included all available data in patient medical records from birth until either the date of data collection or date of death, as applicable. Participants in the SHARE study provided informed consent, and the SHARE study, including this analysis, was approved by the Institutional Review Board of BCH/DFCI. This study followed the Strengthening the Reporting of Observational Studies in Epidemiology (STROBE) reporting guideline.

### Data Collection

At the time of study enrollment in SHARE, participants completed baseline surveys in which data, including race and ethnicity (self-reported), were collected to allow for comparisons between demographic groups. Medical records were abstracted and linked to the Pediatric Health Information System (Children’s Hospital Association) retrospectively and prospectively, as consented by participants.^11^

Patients within the SHARE database who were cared for at BCH/DFCI were identified, and medical records were reviewed for clinical data not contained in the SHARE database. Abstracted data included a patient’s primary diagnosis and time of diagnosis, date of death as applicable, and all interventions performed from the time of primary diagnosis until the point of data collection. Primary diagnosis was determined via review of patient history and physical notes, initial palliative care consultation notes, and operative reports detailing indications for interventions. Demographic data were obtained from the SHARE database.

Surgical interventions included all procedures and operations performed under sedation or general anesthesia. Interventions performed under local anesthesia were excluded. Surgical intervention types, their indications, and their performing teams were abstracted from operative reports of patient records. Surgical interventions performed during the same encounter were counted as separate interventions when performed by separate teams or performed on different anatomic locations by the same team. Every fifth patient was reviewed by 2 different researchers to facilitate accuracy of medical record abstraction. Differences in abstraction were adjudicated by the principal investigator (D.I.E.).

### Coding Definitions and Development

To accommodate variability in *International Statistical Classification of Diseases and Related Health Problems, Tenth Revision *(*ICD-10*) procedure coding system codes describing similar interventions, each procedure underwent a review and recoding process using standardized terminology. For example, although *gastrostomy tube placement* and *gastrojejunostomy tube placement* are technically different interventions, they were both recoded as *enteral access*. This enabled the aggregation of interventions for analysis purposes.

A complete list of patient *International Statistical Classification of Diseases, Tenth Revision, Clinical Modification *(*ICD-10-CM*) codes obtained from Pediatric Health Information System records was classified into complex chronic condition (CCC)^12^ categories on entry into SHARE.^13^ For this study, a single primary diagnosis category was assigned to each patient and was defined as a patient’s initial serious illness, which was not secondary to or a sequela of any previous serious illness. Primary diagnosis categories were generated inductively by the research team and grouped based on differences in management, illness trajectory, and the predominantly responsible specialty team. For example, if a patient’s initial serious illness was congenital heart disease and the patient was treated primarily by a cardiology team, the primary diagnosis was categorized as cardiovascular. Although each patient with seriously illness is inherently medically complex and cared for by other specialist teams, those teams would manage problems that arose secondarily to the primary diagnosis (for example, respiratory or gastrointestinal dysfunction in the setting of congenital heart disease). A patient may have multiple complex chronic conditions, but each patient has only 1 primary diagnosis.

Goals of intervention categories were adapted from the well-established goals-of-care framework developed by subspecialty palliative care. Goals of intervention, like goals of care, included living as long as possible (life extension), living as well as possible (life enhancement), or a blended approach of living as well as possible for as long as possible (both).^14,15^ Purposes of intervention categories were conceptualized as different from goals to reflect what the intervention was meant to accomplish for a patient. Preliminary categories of intended purposes included diagnostic, curative and repair, temporizing and bridging, and supportive purposes. After electronic health record review was completed, the supportive category was split into traditionally palliative interventions (supportive) and interventions associated with the placement or maintenance of medical technologies (assistive technology). Notably, while an intervention may have a foremost or primary goal, it could also accomplish other goals or purposes. For example, a bridging ventricular assist device may be placed as a temporizing measure for a patient with worsening heart failure. Although it may also provide symptom relief, its primary goal is to bridge the patient to a more definitive therapy. In those cases, our assessments were of the primary goal of the intervention from the perspective of the team performing or recommending it, as determined by a combination of the performing team’s operative report and patient’s associated clinical context. Definitions developed for each goal and purpose are as follows:

Goals: a surgical intervention performed with the primary goal of helping a patient:
  Life enhancing: feel better.
  Life extending: live longer.
  Both: live better for longer.
  Neither: a surgical intervention in which the primary goal is neither living longer nor feeling better.
Intended purposes: a surgical intervention performed on an elective, urgent, or emergent basis for the primary purpose of:
  Diagnostic: identifying an unknown illness or problem.
  Curative or repair: curing or repairing an illness or problem.
  Temporizing or bridging: temporizing or bridging a patient with an illness or problem to another definitive therapy.
  Supportive: supportive care or symptom relief not related to medical technology.
  Assistive technology: either temporary or permanent placement or maintenance of supportive medical technologies.
  Note: the illness or problem being addressed may be related or unrelated to the patient’s primary diagnosis.

### Statistical Analysis

Our analyses described goals and intended purposes of surgical interventions performed after the onset of the primary diagnosis. We summarized categorical variables using relative frequencies and continuous variables using means, medians, and IQRs. All analyses were 2-tailed with a significance level of *P* < .05 and were performed using Stata statistical software version 17.0 (StataCorp). Data were analyzed in September 2023.

## Results

This cohort consisted of 197 youths (mean [SD] age at palliative care start, 8.01 [7.53] years; 108 male [54.8%]; 6 Asian [3.0%], 12 Black [6.1%], 129 White [65.5%], and 16 with >1 race [8.1%]; 27 Hispanic [13.7%] and 142 not Hispanic [72.1%]) (Table 1). The most common primary diagnoses were cardiovascular (48 patients [24.3%]), neurologic (40 patients [20.3%]), and solid tumor or central nervous system (CNS) cancers (28 patients [14.2%]). Youths had mean (SD) of 5.3 (2.08) complex chronic conditions. PPC consultation began a mean (SD) 55.6 (75.3) months after primary diagnosis. Within this cohort, 121 youths (61.4%) were alive at the time of our analysis. In total, 189 youths (95.9%) underwent 3331 interventions. Patients underwent mean (SD) of 17.5 (16.3) interventions and a median (IQR) 13 (5-22) interventions from the time of diagnosis, and half of patients underwent 13 or more interventions.

**Table 1.** Study Population Characteristics

Characteristic	Patients, No. (%) (N = 197)
Underwent surgical intervention	189 (95.9)
Age at PPC start, mean (SD), y	8.01 (7.53)
Sex	
Female	89 (45.2)
Male	108 (54.8)
Race	
American Indian or Alaska Native	2 (1.0)
Asian	6 (3.0)
Black or African American	12 (6.1)
White	129 (65.4)
>1 Race	16 (8.1)
Unknown or not reported	32 (16.2)
Ethnicity	
Hispanic	27 (13.7)
Not Hispanic	142 (72.1)
Unknown or not reported	28 (14.2)
Primary diagnosis	
Cardiovascular	48 (24.3)
Gastrointestinal or genitourinary	10 (5.1)
Genetic or metabolic	27 (13.7)
Hematologic	14 (7.1)
Cancer (solid or CNS)	28 (14.2)
Neurologic	40 (20.3)
Prematurity or neonatal	21 (10.7)
Respiratory	9 (4.6)
No. of CCCs, mean (SD)	5.30 (2.08)
Time from diagnosis to PPC start, mean (SD), mo	55.62 (75.25)
Mortality status	
Alive	121 (61.4)
Deceased	76 (38.6)
Total surgical interventions, No.	3331
Surgical interventions per patient	
Mean (SD)	17.5 (16.3)
Median (IQR)	13 (5-22)

Abbreviations: CCC, complex chronic condition; CNS, central nervous system; PPC, pediatric palliative care.

### Common Surgical Interventions and Intended Purposes

The most commonly performed surgical interventions (Table 2) were intrathecal chemotherapy administration (277 interventions, including 235 interventions [84.6%] with a curative or repair purpose), central venous access (253 interventions, including 251 interventions [99.2%] with an assistive technology purpose), upper endoscopy (250 interventions, including 238 interventions [95.2%] with a diagnostic purpose), bronchoscopy (202 interventions, including 196 interventions [97.0%] with a diagnostic purpose), and phenol, botulinum toxin, or steroid injections (176 interventions, all of which [100%] were for supportive purposes). Biopsies, enteral access (initial placement and exchanges or revisions), endoscopy, and dental rehabilitation were also frequently performed. More invasive interventions, such as complex congenital cardiac repairs (63 interventions, all of which [100%] were for curative or repair purposes) and related operations (41 mediastinal exploration or washout interventions, all of which [100%] were for curative or repair purposes and 25 chest closure interventions, all of which [100%] were for curative or repair purposes), oncologic resection (42 interventions [including 29 interventions [69.0%] for curative or repair purposes), and complex vascular repair (29 interventions, including 28 interventions [96.6%] for curative or repair purposes), were less common but still among the top 25 most frequent surgical interventions.

**Table 2.** Most Common Surgical Interventions

Rank	Interventions						
	Type	Total, No.	No. %				
			Assistive technology	Curative or repair	Diagnostic	Supportive	Temporizing
1	Intrathecal chemotherapy administration	277	0	235 (84.6)	1 (0.4)	41 (15.0)	0
2	Central venous access	253	251 (99.2)	0	1 (0.4)	1 (0.4)	0
3	Upper endoscopy with or without biopsy	250	0	11 (4.4)	238 (95.2)	0	1 (0.4)
4	Bronchoscopy	202	0	5 (2.5)	196 (97.0)	0	1 (0.5)
5	Phenol, botulinum toxin, or steroid injection	176	0	0	0	176 (100)	0
6	Bone marrow aspirate or biopsy	164	0	3 (1.9)	155 (94.4)	6 (3.7)	0
7	Lower endoscopy with or without biopsy	117	0	1 (0.9)	116 (99.1)	0	0
8	Enteral access (initial)	110	110 (100)	0	0	0	0
9	Enteral access exchange, revision, or replacement	109	108 (99.1)	0	1 (0.9)	0	0
10	Laryngoscopy or bronchoscopy	105	0	2 (1.9)	103 (98.1)	0	0
11	Cardiac catheterization with or without intervention	98	0	25 (25.5)	72 (73.5)	0	1 (1.0)
12	Dental care or rehabilitation	69	0	69 (100)	0	0	0
13	Complex congenital cardiac repair or palliation	63	0	63 (100)	0	0	0
14	Lumbar puncture	60	0	3 (5.0)	57 (95.0)	0	0
15	Tympanostomy and tube placement or exchange	57	2 (3.5)	0	0	55 (96.5)	0
16	Exam under anesthesia (ENT)	49	0	0	23 (46.9)	26 (53.1)	0
17	CSF diversion (VP, VA, or subgaleal shunt or ventriculostomy) exchange, revision, or replacement	48	45 (93.8)	1 (2.1)	2 (4.2)	0	0
18	Tracheostomy (initial)	47	47 (100)	0	0	0	0
19	Oncologic resection	42	0	29 (69.0)	0	13 (31.0)	0
20	Mediastinal washout or exploration	41	0	41 (100)	0	0	0
21	Tracheostomy tube exchange or replacement	35	35 (100)	0	0	0	0
22	Complex vascular repair or reconstruction	29	1 (3.4)	28 (96.6)	0	0	0
23	Tonsillectomy or adenoidectomy	26	0	26 (100)	0	0	0
24	Chest closure	25	0	25 (100)	0	0	0
25	Muscular tenotomy or lengthening (multiple sites)	23	0	0	0	23 (100)	0

Abbreviations: CSF, cerebrospinal fluid; ENT, ear, nose, and throat; VA, ventriculoatrial; VP, ventriculoperitoneal.

### Goals and Purposes of Surgical Intervention

Of 3331 surgical interventions (Table 3), there were 878 interventions (26.5%) with the goal of life extension (ie, to live as long as possible), 1229 interventions (37.1%) for life enhancement (ie, to live well as possible), and 79 interventions (2.4%) with the goal of life extension and life enhancement (ie, to live as well as possible for as long as possible). The remaining 1130 interventions (34.1%) had neither goal.

**Table 3.** Primary Goals and Purposes of Surgical Intervention

	Interventions, No. (%)
Intended goal	
Life extending	878 (26.5)
Life enhancing	1229 (37.1)
Both life extending and life enhancing	79 (2.4)
Neither life extending nor life enhancing	1130 (34.1)
Intended purpose	
Diagnostic	1092 (32.9)
Curative or repair	1055 (31.8)
Temporizing or bridging	39 (1.2)
Supportive	434 (13.1)
Assistive technology	696 (21.0)

Most surgical interventions were performed for the intended purpose of diagnosis (1092 interventions [32.9%]) or cure or repair (1055 interventions [31.8%]). Interventions for the purpose of placing or maintaining assistive technology were the next most commonly performed (696 interventions [21.0%]), followed by those that were supportive (434 interventions [13.1%]) or temporizing (39 interventions [1.2%]). Interventions performed for diagnostic purposes were distributed relatively evenly across several primary diagnosis categories (genetic, then cardiovascular, neurologic, hematologic, and prematurity), and similar patterns were observed for assistive technology (Table 4). Patients with cardiovascular disease or cancers (hematologic, solid tumor, or CNS) constituted approximately half (592 patients [56.1%]) of those undergoing curative or repair interventions, whereas youths with neurologic or genetic conditions constituted approximately half (244 patients [56.2%]) of those undergoing supportive interventions. Temporizing interventions were a small proportion of those performed, with most among patients with cardiovascular diagnoses (20 patients [51.3%]).

**Table 4.** Distribution of Intended Purposes by Primary Diagnosis

Purpose	Interventions, No. (%)^a^				
	Assistive technology (n = 696)	Curative or repair (n = 1055)	Diagnostic (n = 1092)	Supportive (n = 434)	Temporizing (n = 39)
Patients, No.	157	170	162	67	67
Primary diagnosis rank					
1	Genetic: 149 (21.4)	Cardiovascular: 281 (26.6)	Genetic: 226 (20.7)	Neurologic: 169 (38.9)	Cardiovascular: 20 (51.3)
2	Neurologic: 120 (17.2)	Hematologic: 151 (14.3)	Cardiovascular: 183 (16.8)	Genetic: 75 (17.3)	Neurologic: 6 (15.4)
3	Cancer (solid or CNS): 115 (16.5)	Cancer (solid or CNS): 160 (15.2)	Neurologic: 142 (13.0)	Cardiovascular: 51 (11.8)	Prematurity: 5 (12.8)
4	Cardiovascular: 101 (14.5)	Genetic: 127 (12.0)	Hematologic: 132 (12.1)	Prematurity: 43 (9.9)	Hematologic: 5 (12.8)
5	Prematurity: 87 (12.5)	Neurologic: 126 (11.9)	Prematurity: 111 (10.2)	Hematologic: 42 (9.7)	Other (including genetic, GI or GU, and respiratory): 3 (7.7)

Abbreviations: CNS, central nervous system; GI, gastrointestinal; GU, genitourinary.

^a^ Percentages reflect the proportion of interventions by primary diagnosis type with each purpose of intervention.

### Purposes of Surgical Intervention Over Time Across Primary Diagnoses

The Figure visually represents the prevalence of intended purposes of surgical interventions over time stratified by primary diagnosis category. Among patients with cardiovascular diagnoses, interventions for cure or repair were most frequent in the early postdiagnosis period, with a smaller early predominance of diagnostic and assistive technology interventions. Patients with prematurity had similar early postdiagnosis cure or repair interventions, although diagnostic and assistive technology interventions were close in number early and peaked to a smaller degree at approximately 10 years after diagnosis. Youths with cancers, including hematologic, solid tumor, and CNS neoplasms, also had early postdiagnosis interventions for the purposes of cure or repair, with a shift to predominantly diagnostic interventions. Youths with solid tumor or CNS cancers also had numerous early postdiagnosis interventions for placement and maintenance of assistive technology. For youths with the previously listed diagnoses, PPC started at mean of within 2.5 years after diagnosis and generally after most interventions. Youths with genetic or neurologic diagnoses had interventions for varied purposes over time, with a predominance in diagnostic and assistive technology interventions; supportive interventions were also frequent in the later years of youths with neurologic diagnoses. Youths with gastrointestinal or genitourinary (GI/GU) and respiratory diagnoses had a predominance of diagnostic interventions. However, these were most frequent in the early postdiagnosis period for youths with respiratory diagnoses and more consistent over time, with interspersed cure and repair interventions and assistive technology for GI/GU diagnoses. Youths with genetic, neurologic, GI/GU, and respiratory diagnoses also had later PPC initiation, with mean (SD) periods ranging from 5.0 (6.6) years after a genetic diagnosis to 11.9 (9.3) years after a respiratory diagnosis.

**Figure.**
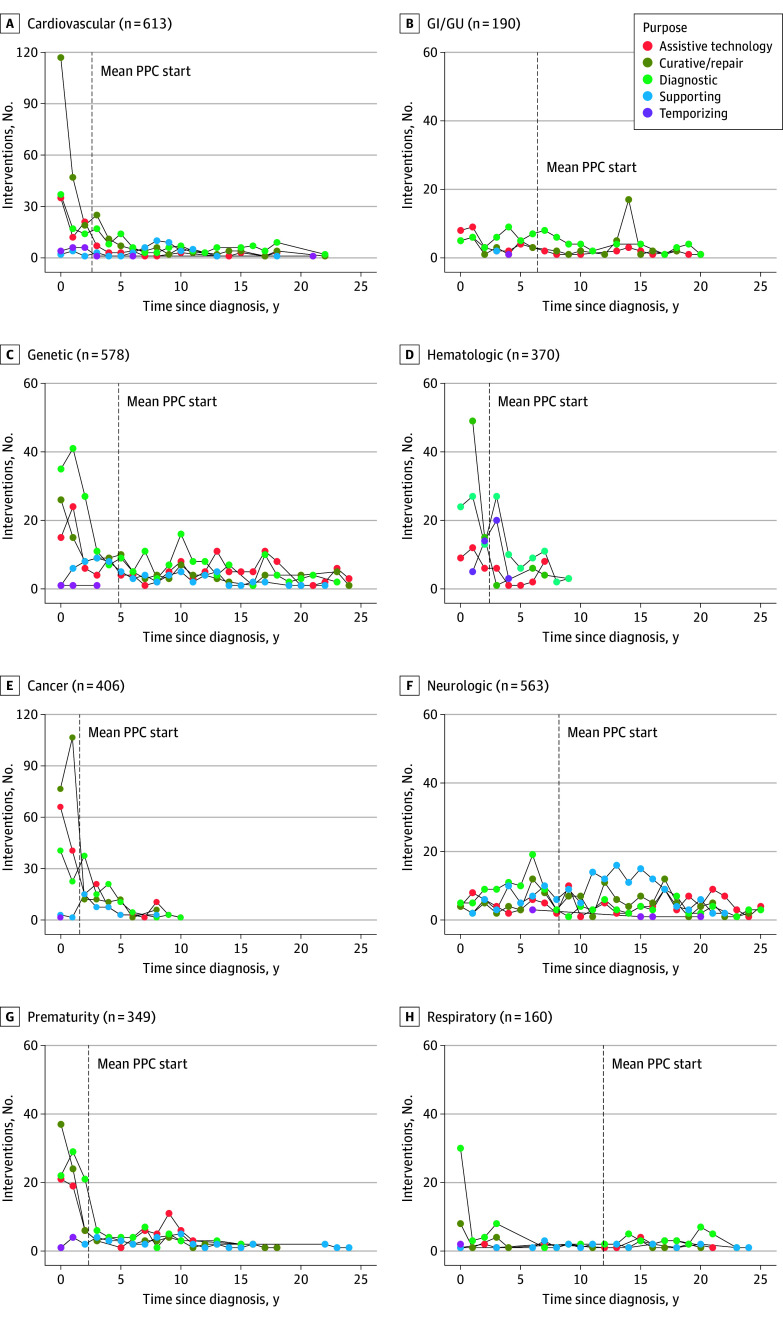
Distribution of Surgical Intervention by Purpose Over Time GI indicates gastrointestinal; GU, genitourinary; PPC, pediatric palliative care.

## Discussion

To our knowledge, this cohort study is the first study that categorizes the goals and intended purposes of surgical intervention in youths with serious illness. Consistent with prior studies, we found that among youths with seriously illness receiving PPC, nearly all patients (95.9%) underwent surgical intervention and half underwent 13 interventions or more from the time of primary diagnosis. Unique to this study,^5^ we found that goals of interventions were relatively balanced among life extension, life enhancement, and neither goal. Intended purposes of intervention, however, were skewed more toward diagnosis, cure and repair, and assistive technology, with fewer interventions performed for supportive purposes and very few as temporizing interventions.

### Goals and Intended Purposes of Surgical Intervention by Serious Illness and Over Time

Purposes of surgical intervention were not evenly distributed across subtypes of serious illness. Youths with more acutely life-threatening illnesses, including cardiovascular disease and cancers, underwent most curative or repair and temporizing interventions, whereas youths with more chronic, life-altering and life-limiting illnesses,^16,17^ such as genetic and neurologic primary diagnoses, underwent most supportive interventions. Diagnostic interventions and those to place or maintain assistive technology were more evenly distributed.

These patterns also changed over time. Patients with acutely life-threatening conditions (ie, cardiovascular primary diagnoses, cancers, and prematurity) tended to undergo interventions for cure or repair in the early postdiagnosis period, with a shift over time to more diagnostic interventions and assistive technology. By contrast, youths with chronic, life-altering, and life-limiting illness (ie, genetic and neurologic diagnoses) tended to undergo interventions for varied purposes over time, with a predominance of diagnostic interventions, assistive technology, and, to a lesser degree, supportive interventions.

The differing patterns of surgical intervention trajectories (including intervention purposes) that emerged between different primary diagnoses and different types of serious illness (ie, acutely life-limiting vs chronic life-limiting or life-altering illnesses) were likely associated with the natural history of disease trajectories. These natural histories may in turn inform team and family goals of care and in part shape what kinds of surgical interventions are recommended and chosen. As an example, it may be that youths with acutely life-threatening illnesses for which cure or repair is possible undergo more curative interventions because these treatments are available and clinically reasonable, offered by teams, and ultimately goal concordant for families pursuing cure. These associations among patient illness trajectory (ie, what is most likely), goals and purposes of intervention (ie, what is possible), clinician recommendations (ie, what the teams recommend), and caregiver decision-making (ie, what is pursued) comprise an area in need of further exploration, as these are the elements that need to be aligned to facilitate truly goal-concordant care.

### Goal-Grounded Language for Goal-Concordant Care

The intended purpose of surgical intervention, or what the intervention is intended to accomplish, can be mapped onto the intervention’s goal as follows (and as diagrammed in the eFigure in Supplement 1). An intervention being performed for diagnostic purposes is neither life extending nor life enhancing given that it is not intended to address but rather to identify a clinical problem. Interventions involving assistive technology, which were commonly performed in this population, could be done with the goal of life extension or life enhancement. For example, cerebrospinal fluid diversion may be recommended for imminently life-threatening ventriculomegaly precipitating herniation (ie, the primary goal of life extension) or symptomatic but slowly progressive hydrocephalus (ie, the primary goal of life enhancement). Interventions performed for cure or repair and temporizing or bridging are inherently life extending, whereas supportive interventions are inherently life enhancing.

Goal-concordant care requires that an intervention’s primary goal (as opposed to potential secondary benefit) as understood by the team performing or recommending aligns with the primary goal of a patient and family and that teams assess these goals on their own or with the support of subspecialty palliative care at each decision-making junction given that family goals change over the course of a youth’s illness.^18^ The preliminary framework that emerged from our findings requires further development to ensure that it optimally encapsulates goals and purposes of surgical intervention in youths with serious illness prior to implementation. However, our intention in devising a novel framework is to suggest language, for which surgeons have indicated a need,^19^ around surgical intervention that mirrors the language of goals of care and intended purposes that can be mapped onto those goals. Sample language for how the goals and purposes of intervention might be integrated into conversation is as follows:

“[Surgical intervention] is being considered for your child because they have been diagnosed with [condition] OR because there is concern for [undiagnosed condition]. This intervention(s) is a [diagnostic/curative/temporizing/supportive/medical technology] one, meaning we intend to [identify/repair/bridge/relieve the symptoms of/place a device to support your child through] what is going on. Our main goal is for [surgical intervention] to help your child [live longer/feel better/both] [though it may also help them live longer/feel better].”

### Limitations

This study has several limitations. Despite a robust study sample, our findings are limited by use of a cohort from a single institution in which all patients were receiving palliative care such that the trajectories and diagnosis-specific patterns of surgical intervention may not be generalizable to youths with serious illness more broadly. Furthermore, purposes of surgical intervention our team generated may be too broad or too narrow, and even within our categories, there could be variation in how clinicians classify interventions. Although we endorse this framework as described, what is most fundamental to goal concordance is that that goals and purpose of intervention be determined in conversation with surgical and medical teams and aligned with what have been assessed to be patient and family goals.

## Conclusions

In this cohort study of youths with serious illness receiving palliative care services, almost all individuals underwent surgical interventions, with half undergoing 13 or more such interventions from the time of diagnosis. Youths with more acute, life-threatening illnesses underwent most curative or repair interventions, particularly in the early postdiagnosis period. Youths with more chronic, life-altering, and life-limiting illnesses underwent most supportive interventions. We present a preliminary framework that facilitates communication about surgical intervention in terms of goals and intended purposes that map onto patients’ larger goals of care. Future directions include refining and testing this framework to ultimately promote goal-concordant, high-quality care for youths with serious illness.

## Notes

Supplement 1. **eFigure.** Goals and Purposes of Surgical Intervention Framework

Supplement 2.
Data Sharing Statement
