# Supplementary material for: Goals of Surgical Interventions in Youths Receiving Palliative Care
Source: JAMA Netw Open. 2024 Nov 8;7(11):e2444072. doi: 10.1001/jamanetworkopen.2024.44072 (PMC11549654; doi:10.1001/jamanetworkopen.2024.44072)
Supplement: Supplement 1. — eFigure. Goals and Purposes of Surgical Intervention Framework [file jamanetwopen-e2444072-s001.pdf]

Supplemental Online Content

Ellis DI, Chen L, Gordon Wexler S, et al. Goals of surgical interventions in youths receiving palliative care. *JAMA Netw Open.* 2024;7(11):e2444072.
doi:10.1001/jamanetworkopen.2024.44072

eFigure. Goals and Purposes of Surgical Intervention Framework

This supplemental material has been provided by the authors to give readers additional information about their work.

© 2024 Ellis DI et al. *JAMA Network Open*.

E-Figure 1: Goals and Purposes of Surgical Intervention Framework

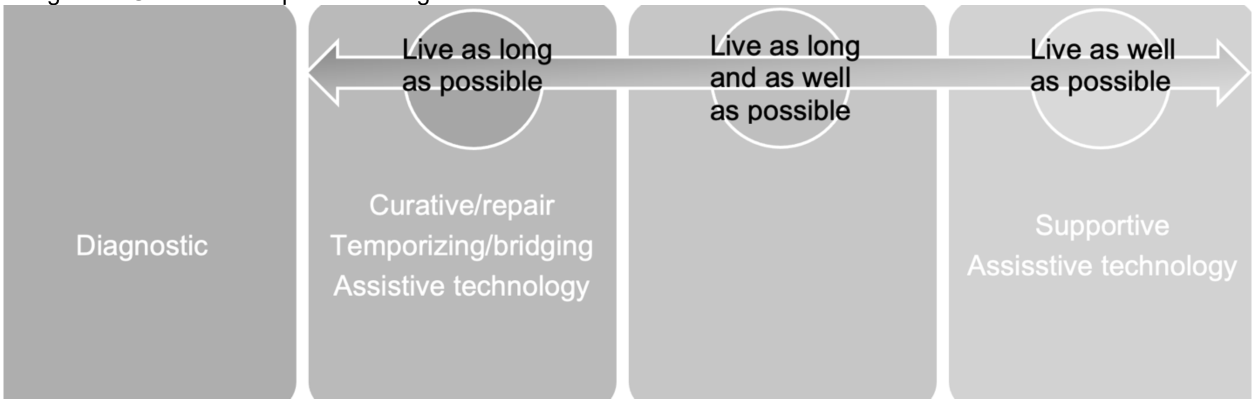

© 2024 Ellis DI et al. *JAMA Network Open*.
