# Supplementary material for: Goals of Surgical Interventions in Youths Receiving Palliative Care
Source: JAMA Netw Open. 2024 Nov 8;7(11):e2444072. doi: 10.1001/jamanetworkopen.2024.44072 (PMC11549654; doi:10.1001/jamanetworkopen.2024.44072)
Supplement: Supplement 2. — Data Sharing Statement [file jamanetwopen-e2444072-s002.pdf]

Data Sharing Statement

Ellis. Goals of Surgical Interventions in Youths Receiving Palliative Care. *JAMA Netw Open*. Published online November 8, 2024. doi:10.1001/jamanetworkopen.2024.44072

Data
Data available: No
